# Health literacy among older adults is associated with their 10-years’ cognitive functioning and decline - the Doetinchem Cohort Study

**DOI:** 10.1186/s12877-018-0766-7

**Published:** 2018-03-20

**Authors:** Bas Geboers, Ellen Uiters, Sijmen A. Reijneveld, Carel J. M. Jansen, Josué Almansa, Astrid C. J. Nooyens, W. M. Monique Verschuren, Andrea F. de Winter, H. Susan J. Picavet

**Affiliations:** 1 Department of Health Sciences, University Medical Center Groningen, University of Groningen, Hanzeplein 1, FA10, P.O. Box 30.001, 9700 RB Groningen, the Netherlands; 2 Centre for Nutrition, Prevention and Health Services, National Institute for Public Health and the Environment (RIVM), Bilthoven, the Netherlands; 3 Department of Communication and Information Studies, Faculty of Arts, University of Groningen, Groningen, the Netherlands; 4 Julius Center for Health Sciences and Primary Care, University Medical Center Utrecht, Utrecht, the Netherlands

**Keywords:** Health literacy, Cognitive functioning, Cognitive decline, Older adults, Longitudinal

## Abstract

**Background:**

Many older adults have low levels of health literacy which affects their ability to participate optimally in healthcare. It is unclear how cognitive decline contributes to health literacy. To study this, longitudinal data are needed. The aim of this study was therefore to assess the associations of cognitive functioning and 10-years’ cognitive decline with health literacy in older adults.

**Methods:**

Data from 988 participants (mean age = 65.3) of the Doetinchem Cohort Study were analyzed. Health literacy was measured by the Brief Health Literacy Screening. Memory, mental flexibility, information processing speed, and global cognitive functioning were assessed at the same time as health literacy and also 10 years earlier. Logistic regression analyses were performed, adjusted for age, gender, and educational level.

**Results:**

Higher scores on tests in all cognitive domains were associated with a lower likelihood of having low health literacy after adjustment for confounders (all ORs < 0.70, *p*-values<.001). Similar associations were found for past cognitive functioning (all ORs < 0.75, *p*-values<.05). Before adjustment, stronger cognitive decline was associated with a greater likelihood of having low health literacy (all ORs > 1.37, *p*-values<.05). These associations lost significance after adjustment for educational level, except for the association of memory decline (OR = 1.40, *p* = .023, 95% CI: 1.05 to 1.88).

**Conclusion:**

Older adults with poorer cognitive functioning and stronger cognitive decline are at risk for having low health literacy, which can affect their abilities to promote health and self-manage disease. Low health literacy and declining cognitive functioning might be a barrier for person-centered care, even in relatively young older adults.

## Background

Many older adults in developed countries have low health literacy [1, 2], defined as “the degree to which people are able to access, understand, appraise and communicate information to engage with the demands of different health contexts to promote and maintain health across the life-course [3].” The high prevalence of low health literacy among older adults – up to 59% [1] - is problematic as this population is in greater need of health information, for example due to the high prevalence of chronic diseases in this group. Low health literacy among older adults is associated with various undesirable health outcomes like poorer self-rated health [4] and higher mortality [5]. It may also affect older adults’ ability to benefit optimally from healthcare services, in which patients are nowadays expected participate actively.

Poor cognitive functioning or cognitive decline may affect the health literacy level of older adults [6]. Lower educational level contributes to low health literacy as well [1], but this does not explain why health literacy tends to gradually decline over time in older adults [7, 8]. Specific domains of cognitive functioning that might play a role in the health literacy of older adults are memory, information processing speed, and mental flexibility. Cross-sectional studies have shown that poor cognitive functioning is related to low health literacy in older adults, for example among patients with diabetes [9]. Other studies suggest that poor cognitive functioning partially explains the association between low health literacy and undesirable health outcomes like poor physical health [10]. However, evidence lacks about how a decline in cognitive functioning affects health literacy at an older age. In order to answer such research questions, longitudinal data is needed.

Current evidence on the longitudinal association of cognitive decline with health literacy is very limited. One study has shown that cognitive abilities at age 11 are positively associated with health literacy at age 67 [11], but this does not regard cognitive decline. Only two studies addressed associations of cognitive decline with low health literacy [12] and health literacy decline [7] but these regarded a relatively short follow-up time, i.e. less than 6 years. Evidence on the associations of cognitive decline and health literacy during longer follow-up periods fully lacks.

The aim of this study is to assess the associations of cognitive functioning and 10-years’ cognitive decline with health literacy in older adults, by taking into account global cognitive functioning and the domains of memory, mental flexibility, and information processing speed.

## Methods

### Design, setting, and study population

Data from the prospective Doetinchem Cohort Study (DCS) were used for our analyses [13]. All participants are inhabitants of Doetinchem, a city in the East of the Netherlands (population around 57,000). The general aim is to study the impact of lifestyle and biological risk factors on health. The first round of data collection took place in 1987–1991 and included 7768 participants aged 20–59 years (response rate: 62%). Follow-up rounds took place every 5 years, with response rates of > 75%. The fifth round (2008–2012) included 4018 participants (51.7% of the primary sample). The sixth round is ongoing. The DCS was conducted according to the guidelines laid down in the Helsinki Declaration, with all procedures approved by the external Medical Ethics Committees. Written informed consent was obtained from all participants before the start of the study.

For our analyses, we included participants whose data from the sixth round were available by February 2016 (*n* = 1028). We excluded participants who reported to have had a stroke (2.7%, *n* = 28), as strokes are known to have an impact on cognitive functioning. As one of the cognitive tests was language specific, we also excluded participants who moved to the Netherlands after the age of 18 (1.2%, *n* = 12), leaving a set of 988 participants.

Data on cognitive functioning were collected from round three onwards from participants above the age of 45. We used data from round six for current cognitive functioning and from round four for past cognitive functioning. In this paper, round four will be described as the first measurement and round six will be described as the second measurement. The follow-up time between these measurements was 10 years (mean = 9.77, SD = 0.18). Cognitive data in the first measurement were collected from 737 participants (74.6% of our set). This lower number is the result of some participants not having reached the age of 45 when this measurement took place.

### Measures

We used data on health literacy, cognitive functioning, and covariates.

#### Health literacy

Health literacy was measured during the second measurement by the validated Brief Health Literacy Screening [14, 15]. It consists of three items:

1. “How often do you have someone help you read hospital materials?”
2. “How confident are you filling out medical forms by yourself?”
3. “How often do you have problems learning about your medical condition because of difficulty understanding written information?”

Participants answered these questions on a 5-point scale. We added up the scores of all questions, which led to a continuous scale (3–15). We then categorized participants into groups with high health literacy (13 or higher, 81.8%) and low health literacy (12 or lower, 18.2%), based on previous research [16].

#### Cognitive functioning

Four tests were used to measure cognitive functioning: the 15-word Verbal Learning Test (*VLT*) [17], the Stroop Color Word Test (*SCWT*) [18], the Verbal Fluency Test (*VFT*) [19], and the Letter Digit Substitution Test (*LDST*) [20]. The *VLT* consists of showing the participant 15 monosyllabic words three times, with a free recall test after every presentation and an additional free recall test after a 15 min delay. Scores are determined by the number of words that are correctly recalled. The *SCWT* consists of three subtasks. In the first subtask, participants are asked to read 40 written color names. In the second subtask, they are asked to name the color of 40 colored patches. The third subtask consists of naming the ink color in which 40 color names are shown incongruously (e.g., the word “red” shown in green). Scores are determined by the time it took the participant to complete each task. Scores of the *SCWT* were reversed, whereby a higher score indicated a better performance; scores were then normalized by log transformation, because of the skewed distribution. The *VFT* consists of asking the participant to name as many animals as possible within 1 minute. The *LDST* consists of a sheet of paper containing a table in which nine letters are matched with a digit (1 to 9) and a separate list of letters. The participant is asked to match the letters in the list with the corresponding digits as quickly as possible. The score is based on the number of letters that were matched correctly. All these tests are known to be sensitive to detect age-related cognitive decline, also in the middle-age range.

Scores on all tests were standardized. Scores from the first measurement were standardized based on the means and standard deviations of the same tests in the second measurement. Scores were then combined into three cognitive domains and a score for global cognitive functioning [21]. The following formulas were used:

$$ {\displaystyle \begin{array}{l}\mathrm{Memory}=\left({\mathrm{VLT}}_{\mathrm{Total}}+{\mathrm{VLT}}_{\mathrm{Maximum}}+{\mathrm{VLT}}_{\mathrm{Delayed}}\right)/3\\ {}\mathrm{Information}\kern0.17em \mathrm{processing}\kern0.17em \mathrm{speed}=\left({\mathrm{Stroop}}_{\mathrm{Color}\ \mathrm{names}}+{\mathrm{Stroop}}_{\mathrm{Color}\ \mathrm{patches}}+\mathrm{LDST}\right)/3\\ {}\mathrm{Mental}\ \mathrm{flexibility}={\mathrm{Stroop}}_{\mathrm{Ink}\ \mathrm{color}}\\ {}\mathrm{Global}\kern0.17em \mathrm{cognitive}\ \mathrm{functioning}=\left({\mathrm{Stroop}}_{\mathrm{Ink}\ \mathrm{color}}+\mathrm{LDST}+{\mathrm{VLT}}_{\mathrm{Total}}+{\mathrm{VLT}}_{\mathrm{Delayed}}+\mathrm{VFT}\right)/5\end{array}} $$

Cognitive decline concerned the domain scores at the second measurement minus the domain scores at the first measurement. A higher score thus indicates stronger cognitive decline.

#### Covariates

Educational level was used as a covariate in our study. It was assessed based on the highest completed level of education in five categories: Very low (elementary school or less), low (lower vocational education), medium (medium general secondary education), high (medium vocational education to pre-university education), and very high (higher vocational education or university). Further potential confounders were age and gender. To control for potential non-linear effects of age, age squared was also used.

### Statistical analyses

First, we compared the age, gender, educational level, and current cognitive functioning of the participants by level of health literacy, by using chi-square tests and independent sample t-tests. We also tested whether cognitive decline had taken place between the two measurement waves by using paired samples t-tests. Second, we assessed the associations of the various cognitive domains (both current and past) with health literacy by using logistic regression analyses, adjusted for covariates, and with health literacy as the outcome variable. Finally, we repeated the analyses with cognitive decline in all domains as the predictor variables, additionally adjusted for past cognitive functioning.

### Sensitivity analyses

We conducted two sensitivity analyses. First, we checked whether the use of an alternative cut-off point for high health literacy would lead to changes in our results. For this purpose, we used a cut-off point of 14 or higher (63.5% high health literacy vs. 81.8% in the main analyses). Second, we checked whether the use of data from a different wave for past cognitive functioning would change our results. For this purpose, we studied cognitive functioning at round three (15 year follow-up time, *n* = 643).

## Results

Characteristics of the sample at the second measurement and their associations with health literacy are presented in Table 1. Participants with low health literacy were older, more often had a (very) low level of education, and had poorer cognitive functioning in all domains. Participants who had also participated in cognitive testing in the first measurement (*n* = 737) had a mean age of 68.5 years at the second measurement, and showed cognitive decline in all domains between the two measurement waves (all *p*-values<.0001).

**Table 1:** Background characteristics and current cognitive functioning of participants by level of health literacy (*n* = 988)

	Health literacy			
	High (81.8%)	Low (18.2%)	Total	*p*
Age (years)	64.9	66.8	65.3	.015
Male (%)	47.4	45.0	47.0	.56
(Very) low-educated (%)	26.3	54.3	31.5	<.001^a^
Cognitive functioning (z-scores)				
Memory	0.09	−0.38	0.00	<.001
Information processing speed	0.09	−0.39	0.00	<.001
Mental flexibility	0.10	−0.47	0.00	<.001
Global cognitive functioning	0.10	−0.39	0.01	<.001

*p*-values are based on chi-square tests and independent samples t-tests. Percentages of missing data were low (all percentages< 3.2%)

^a^*p*-value is based on five levels of education

### Associations between cognitive functioning and health literacy

Associations of current and past cognitive functioning with health literacy are shown in Table 2. Poorer current cognitive functioning in all domains was significantly associated with low health literacy after adjustment for age and gender. These associations weakened after adjustment for educational level, but they all remained statistically significant. The same pattern of results was found for the associations between past cognitive functioning and health literacy.

**Table 2:** Likelihood of having low health literacy per one standard deviation better cognitive functioning among older adults, in odds ratios (and 95% confidence intervals)

	Low health literacy (vs. high)	
	Model 1^a^	Model 2^b^
Current cognitive functioning (in z-scores)		
Memory (*n* = 944)	0.58*** (0.48–0.71)	0.69*** (0.56–0.85)
Information processing speed (*n* = 947)	0.50*** (0.40–0.63)	0.66*** (0.52–0.84)
Mental flexibility (*n* = 942)	0.56*** (0.46–0.68)	0.67*** (0.54–0.83)
Global cognitive functioning (*n* = 932)	0.38*** (0.29–0.50)	0.51*** (0.38–0.69)
Past cognitive functioning (in z-scores; measured 10 years earlier)		
Past memory (*n* = 732)	0.55*** (0.43–0.71)	0.71* (0.54–0.93)
Past information processing speed (*n* = 734)	0.49*** (0.37–0.66)	0.71* (0.51–0.97)
Past mental flexibility (*n* = 733)	0.56*** (0.43–0.73)	0.75* (0.56–1.00)
Past global cognitive functioning (*n* = 729)	0.34*** (0.25–0.48)	0.51*** (0.35–0.75)

*p < .05, **p < .01, ***p < .001

^a^Adjusted for age, age squared, and gender

^b^Adjusted for age, age squared, gender, and educational level

### Associations between cognitive decline and health literacy

Associations between cognitive decline and health literacy are presented in Table 3. After adjustment for age, gender, and past cognitive functioning, decline in all cognitive domains was statistically significantly associated with low health literacy. Further adjustment for educational level weakened all associations. Only the association between memory decline and health literacy then remained statistically significant (OR = 1.40, *p* = .023).

**Table 3:** Likelihood of having low health literacy per one standard deviation stronger cognitive decline among older adults, in odds ratios (and 95% confidence intervals)

	Low health literacy (vs. high)		
	Model 1^a^	Model 2^b^	Model 3^c^
Memory decline (*n* = 723)	1.09 (0.85–1.39)	1.50** (1.13–1.99)	1.40* (1.05–1.88)
Information processing speed decline (*n* = 728)	1.27 (0.88–1.83)	1.50* (1.03–2.19)	1.29 (0.87–1.91)
Mental flexibility decline (*n* = 723)	1.19 (0.89–1.60)	1.37* (1.03–1.84)	1.27 (0.93–1.73)
Global cognitive functioning decline (*n* = 712)	1.10 (0.71–1.71)	1.68* (1.05–2.67)	1.54 (0.95–2.51)

*p < .05, **p < .01

Cognitive decline calculated as the difference between current and past cognitive functioning, with a higher score indicating stronger decline

^a^Adjusted for age, age squared, and gender

^b^Adjusted for age, age squared, gender, and past cognitive functioning

^c^Adjusted for age, age squared, gender, past cognitive functioning, and educational level

### Sensitivity analyses

The sensitivity analyses with the alternative cut-off point for low health literacy did not lead to relevant changes in the associations between current and past cognitive functioning and health literacy. The associations between cognitive decline and health literacy before adjustment for educational level were weakened, with only the association between health literacy and memory decline remaining statistically significant (OR = 1.30, *p*-value<.05), and the other associations losing significance (ORs < 1.42, all *p*-values between .055 and .11). After adjustment for educational level, none of the associations reached statistical significance (all ORs < 1.26, *p*-values>.14).

Using data from a different measurement wave for past cognitive functioning (round three) did not change our results regarding the associations of current and past cognitive functioning with health literacy. The only relevant change in the associations between cognitive decline and health literacy was that, after adjustment for educational level, the association between memory decline and health literacy lost significance (OR = 1.26, *p*-value = .15), while the association between mental flexibility decline and health literacy remained statistically significant (OR = 1.36, *p*-value<.05).

## Discussion

In this longitudinal study, we assessed the associations of current and past cognitive functioning and cognitive decline with health literacy in older adults. Our study shows that poorer cognitive functioning in both the present and the past is associated with low health literacy. Stronger cognitive decline is also associated with low health literacy, but after adjustment for educational level, only the association between health literacy and memory decline remains statistically significant.

Our study showed associations between poorer current cognitive functioning and low health literacy among older adults for global cognitive functioning and all its domains (i.e., memory, information processing speed, and mental flexibility). This confirms previous findings that cognitive functioning and health literacy are related [9, 10, 12] and suggests that health literacy in older adults is dependent on a variety of cognitive abilities. The identified associations between poorer past cognitive functioning and low health literacy are in line with the findings of Mõttus et al. [11], who found that cognitive functioning in childhood is associated with health literacy at an older age. Our findings support their conclusion that health literacy reflects lifelong general cognitive ability [11]. All associations weakened after adjustment for educational level, which could be explained by the associations of educational level with both health literacy [1] and cognitive functioning [22] found in earlier studies. However, in our study, the association between cognitive functioning and health literacy is for the most part independent of educational level, probably because educational level is determined not only by capabilities, but also by opportunities.

To our knowledge, our study is the first to suggest that associations between cognitive decline and low health literacy already exist in relatively young older adults. In our study, the associations between cognitive decline and health literacy seem to be partially independent of educational level. This may partially explain why health literacy gradually declines over time among older adults [7, 8]. This issue requires further study.

The relationships of cognitive functioning and cognitive decline with health literacy might also go beyond a one-way cause-and-effect relationship in which cognition affects the health literacy of older adults. Among older adults, having a low level of health literacy might also lead to poorer cognitive functioning. For example, older adults with low health literacy might engage in less physical activity [23], which might then contribute to lower cognitive health [24]. A degree of conceptual overlap may also exist between cognitive functioning and health literacy. However, empirical studies show that cognitive functioning and health literacy are separate constructs that have independent effects on various outcomes [25, 26].

### Strengths and limitations

This was the first longitudinal study on cognitive functioning and health literacy with a long follow-up time of 10 years. An important strength of our study was the use of a community-based sample. The longitudinal nature of the dataset used allowed us to study past cognitive functioning and cognitive decline. Also, we used a set of sensitive instruments to measure cognitive functioning.

Our study also had some limitations. First, our results may be affected by selective response and drop-outs. However, response rates were high in all rounds of the DCS (> 75%), limiting the potential for this bias. Additionally, we studied associations, which are much less affected by such bias than prevalences [27]. Second, we used a self-report instrument to measure health literacy. This may have led to an underestimation of some associations, as people may not always be aware of their health literacy limitations. However, the Brief Health Literacy Screening we used has been validated [14, 15] and is frequently used in research [28, 29].

### Implications

Many healthcare services are increasingly shifting towards a more person-centered approach [30]. Person-centered care is defined as “an approach to care that consciously adopts the perspectives of individuals, families and communities, and sees them as participants as well as beneficiaries of trusted health systems that respond to their needs and preferences in humane and holistic ways” [30]. This approach to care assumes that patients actively take part in their own care and make their own decisions with regard to treatment options. However, low health literacy and declining cognitive functioning in older adults might be important barriers for person-centered care. It is therefore important that healthcare professionals involved in prevention and care for older adults are aware of the important impact of cognitive functioning on health literacy. Proactive planning of care is essential for older adults with both low health literacy and declining cognitive functioning. As cognitive decline is even associated with low health literacy in relatively young older adults, health professionals should be aware that early identification of the most vulnerable group is especially important.

The associations between poor cognitive functioning and low health literacy also suggest that interventions to mitigate the negative impacts of low health literacy should focus on lowering the cognitive demands of health information. This may include strategies like the use of plain language in health documents, but also the use of spoken animations [31] and teach-back methods [32]. It might be important to address both cognition and health literacy in order to optimize person-centered prevention and health care.

To further disentangle the intricate relationship between cognitive functioning and health literacy, future studies should measure both factors repeatedly over the course of several years. Additionally, such studies could examine the potential confounding or mediating role of health status in these relations. Future research should also focus on the possibility of limiting health literacy decline among older adults by using cognitive interventions, such as cognitive training [33].

## Conclusion

Our findings show that, in older adults, poor cognitive functioning in both the present and the past is associated with low health literacy. This holds for memory, information processing speed, and mental flexibility, and most strongly for global cognitive functioning. Further, even in relatively young older adults, cognitive decline is associated with low health literacy, also when taking educational level into account. This has implications for prevention and care regarding older adults with low cognitive functioning.

## Abbreviations

DCS: Doetinchem Cohort Study
LDST: Letter digit substitution test
SCWT: Stroop color word test
VFT: Verbal fluency test
VLT: Verbal learning test
